# Exploring Coagulation Abnormalities and Functional Impairment in Long COVID: Relevance to Primary Care Practice

**DOI:** 10.7759/cureus.109403

**Published:** 2026-05-21

**Authors:** Rama Krishna Prasad Chikkala, Sandeep Garre, A Chaitanya Pratyusha, Syama Sundar Ayya, Kalyani Sangineni, Sneha Gogula

**Affiliations:** 1 Anaesthesiology, All India Institute of Medical Sciences, Bibinagar, Bibinagar, IND

**Keywords:** 6-minute walk test, coagulation, d-dimer, fibrinogen, long covid, post-covid sequelae

## Abstract

Background

The long-term sequelae of COVID-19, collectively termed long COVID, manifest as persistent symptoms that may be driven by ongoing coagulation abnormalities. Early identification, particularly in primary care, requires simple, low-cost tools that do not rely on advanced diagnostics. This study evaluated the usefulness of basic coagulation markers and the Six-Minute Walk Test (6MWT) in assessing symptom severity and functional limitation among individuals with long COVID.

Methodology

A two-phase observational study was conducted from July 2022 to January 2024. Phase I enrolled 197 adults more than six weeks post-COVID-19 who presented with fatigue, breathlessness, reduced exercise tolerance, cough, or musculoskeletal pain. Baseline investigations included C-reactive protein (CRP), prothrombin time (PT)/international normalized ratio (INR), activated partial thromboplastin time (aPTT), D-dimer, fibrinogen, and platelet count, alongside the 6MWT. The primary objective was to evaluate the association between coagulation abnormalities and the severity of long COVID symptoms. Secondary objectives included assessment of functional impairment using the 6MWT and evaluation of persistence of coagulation abnormalities at the three-month follow-up. Patients with abnormal baseline coagulation or inflammatory parameters (n = 63) underwent repeat evaluation after three months.

Results

The study included 197 participants with a mean age of 37.3 ± 11.97 years, of whom 87 (44.2%) were male. Reduced effort tolerance was the most common presenting symptom, observed in 151 (76.7%) patients. At baseline, abnormalities were noted in D-dimer (50 (25.4%)), fibrinogen (43 (21.8%)), CRP (34 (17.3%)), aPTT (23 (11.7%)), PT/INR (21 (10.7%)), and platelet count (10 (5.1%)). Patients with severe symptoms demonstrated significantly higher levels of D-dimer and fibrinogen (p < 0.001 for both). Elevated D-dimer (50 (25.4%)) and fibrinogen (43 (21.8%)) were associated with an increased risk of severe symptomatology, with relative risks of 3.0 and 2.34, respectively. At the three-month follow-up, persistent elevation of D-dimer (32 (50.8%)) and fibrinogen (30 (47.6%)) was observed, indicating sustained coagulation abnormalities in a substantial subset of individuals with long COVID.

Conclusions

This study demonstrates a significant association between elevated D-dimer and fibrinogen levels and symptom severity in patients with long COVID. Simple, accessible tools, particularly basic coagulation tests and the 6MWT, may serve as useful adjunctive assessments for primary care physicians to identify long COVID patients with probable ongoing thrombo-inflammatory activity. Integrating these assessments into routine follow-up may improve early detection, monitoring, and targeted referral.

## Introduction

COVID-19 struck the Indian subcontinent with unparalleled intensity, leading to substantial morbidity and mortality. Among those who survived and were declared cured, a considerable number continued to experience persistent symptoms such as breathlessness, cough, and fatigue. To address this unmet clinical need, a post-COVID-19 clinic was established at our institution to evaluate and manage these individuals.

While many patients recover from acute COVID-19, a meaningful subset develop persistent symptoms, including breathlessness, cough, fatigue, and musculoskeletal pain, which are collectively termed long COVID or post-acute sequelae of COVID-19 [1-3]. In acute COVID-19, coagulopathy, thromboembolism, and microvascular immune‐thrombosis are well documented, and they are associated with disease severity and mortality [4,5]. Furthermore, emerging evidence suggests that patients who experienced more severe acute illness are also more likely to develop persistent symptoms, implying a possible mechanistic link between coagulopathy and long COVID [5].

Given the broad spectrum of long COVID symptoms and the fact that most affected individuals first seek evaluation in primary care settings, it is essential to identify simple, accessible clinical tools to help clinicians identify patients at risk. The Six-Minute Walk Test (6MWT) is a simple, inexpensive, and widely accessible functional assessment tool intended to evaluate exercise limitation and cardiopulmonary reserve. Basic coagulation markers and functional assessments such as the 6MWT are inexpensive, widely available, and feasible for routine use in primary care, yet their utility in evaluating long COVID remains underexplored. Establishing whether coagulation abnormalities are associated with these ongoing symptoms may provide valuable insights into disease mechanisms and guide practical, scalable strategies for early identification and management within primary care.

Against this background, we hypothesized that patients with more severe long COVID symptoms would demonstrate greater derangement in coagulation markers, along with poorer functional performance on the 6MWT. The primary objective of this study was to evaluate the association between coagulation abnormalities and symptom severity in patients with long COVID. Secondary objectives were (i) to assess functional impairment using the 6MWT in patients with long COVID and (ii) to evaluate the persistence of coagulation abnormalities and functional limitation at the three-month follow-up. We defined long COVID as the persistence of symptoms such as fatigue, breathlessness, reduced exercise tolerance, cough, or musculoskeletal pain for more than six weeks following a confirmed COVID-19 infection.

## Materials and methods

Study design and setting

A two-phase observational study was conducted between July 2022 and January 2024. In Phase I, we undertook a cross-sectional assessment of symptomatic post-COVID-19 patients. In Phase II, those with abnormal baseline investigations were followed up at three months for repeat assessments. The study received approval from the Institutional Ethics Committee (AIIMS/BBN/IEC/AUG/2021/83), and the trial was registered in the Clinical Trials Registry - India (CTRI/2022/08/044823).

Study population and sampling

Consecutive patients aged 18 years and above who attended the post-COVID-19 follow-up outpatient clinic at our institute were screened for eligibility. Inclusion criteria comprised a confirmed history of COVID-19, established by reverse transcription polymerase chain reaction or rapid antigen testing, completion of at least six weeks since diagnosis, and the presence of persistent symptoms such as fatigue, breathlessness, reduced exercise tolerance, cough, or musculoskeletal pain. Patients with pre-existing coagulation disorders, those receiving anticoagulant therapy, or individuals with comorbid conditions that could limit exercise tolerance (e.g., ischemic heart disease, congestive heart failure, chronic obstructive pulmonary disease, or joint disorders precluding performance of the 6MWT) were excluded. Written informed consent was obtained from all participants before enrolment.

Data collection

A structured case record form captured demographics, comorbidities, acute illness data (onset, severity, hospitalization, oxygen/ventilator use). Long COVID symptoms such as fatigue, breathlessness, musculoskeletal pain, cough, and reduced exercise tolerance were assessed and documented. Symptoms were graded as significant if they were interfering with their routine daily activities, as judged by the patient, and not significant if not interfering with routine daily activities.

During physical examination, vital signs were recorded, and the 6MWT was performed. The 6MWT was performed on a flat, straight, 30-meter-long walkway. Before commencement of the test, patients were seated and rested for approximately 10 minutes. They were instructed to walk for six minutes with the goal of covering as much distance as possible. Verbal encouragement was provided at one-minute intervals using phrases such as “you are doing well.” Patients were permitted to slow down, stop, and rest during the test if necessary; however, the timer was not interrupted, and the total distance covered at the end of six minutes was recorded as the Six-Minute Walk Test Distance (6MWTD). Oxygen saturation (SpO₂) was measured during the procedure using a finger pulse oximeter. Patients whose 6MWTD was less than 400 meters, or those whose saturation fell by more than 4% from their baseline or an absolute value of <88%, were labelled as 6MWT failed.

Under strict aseptic conditions, blood was collected into an EDTA vacutainer for platelet count; a sodium citrate vacutainer for prothrombin time (PT) with international normalized ratio (INR), activated partial thromboplastin time (aPTT), D-dimer, and fibrinogen; and in a plain vacutainer for C-reactive protein (CRP). Platelet count was analyzed by a HORIBA H-550 automated analyzer, and abnormal results were verified by peripheral smear examination. PT with INR, aPTT, D-dimer, and fibrinogen were analyzed by an ERBA coagulation line ECL-760 fully automated analyzer, and CRP was analyzed by the latex agglutination method.

Interventions and follow-up

Patients received individualized symptomatic management based on clinical presentation. Antitussives were prescribed for persistent cough, analgesics for musculoskeletal pain, and physiotherapy or spirometry was recommended for those with reduced exercise tolerance, where clinically indicated. Management was supportive and individualized. Individuals exhibiting elevated D-dimer or fibrinogen levels were further evaluated for deep vein thrombosis and pulmonary thromboembolism and received anticoagulation in consultation with a physician. The study was observational in design, and treatment efficacy was not evaluated as a predefined endpoint.

All participants were scheduled for a follow-up evaluation after three months. During this visit, clinical history, physical examination, and 6MWT were repeated. Repeat laboratory investigations were performed only for patients with abnormal baseline coagulation or inflammatory markers.

Statistical analysis

Based on a previous study reporting a 25.3% prevalence of D-dimer derangement among patients with long COVID [8], the sample size was calculated using a relative precision of 6% and a 95% confidence interval (CI), yielding a target of 197 participants.

Data were analyzed using SPSS Statistics, version 22.0 (IBM Corp., Armonk, NY, USA). Normality of continuous variables was assessed using the Shapiro-Wilk test. Continuous variables were expressed as mean ± standard deviation (SD) and compared using the independent t-test. Categorical variables were expressed as frequency (percentage) and analyzed using the Pearson chi-square test. Relative risk (RR) and 95% CIs were calculated for associations between symptom severity and abnormal D-dimer or fibrinogen values. A p-value <0.05 was considered statistically significant.

## Results

**Figure 1:**
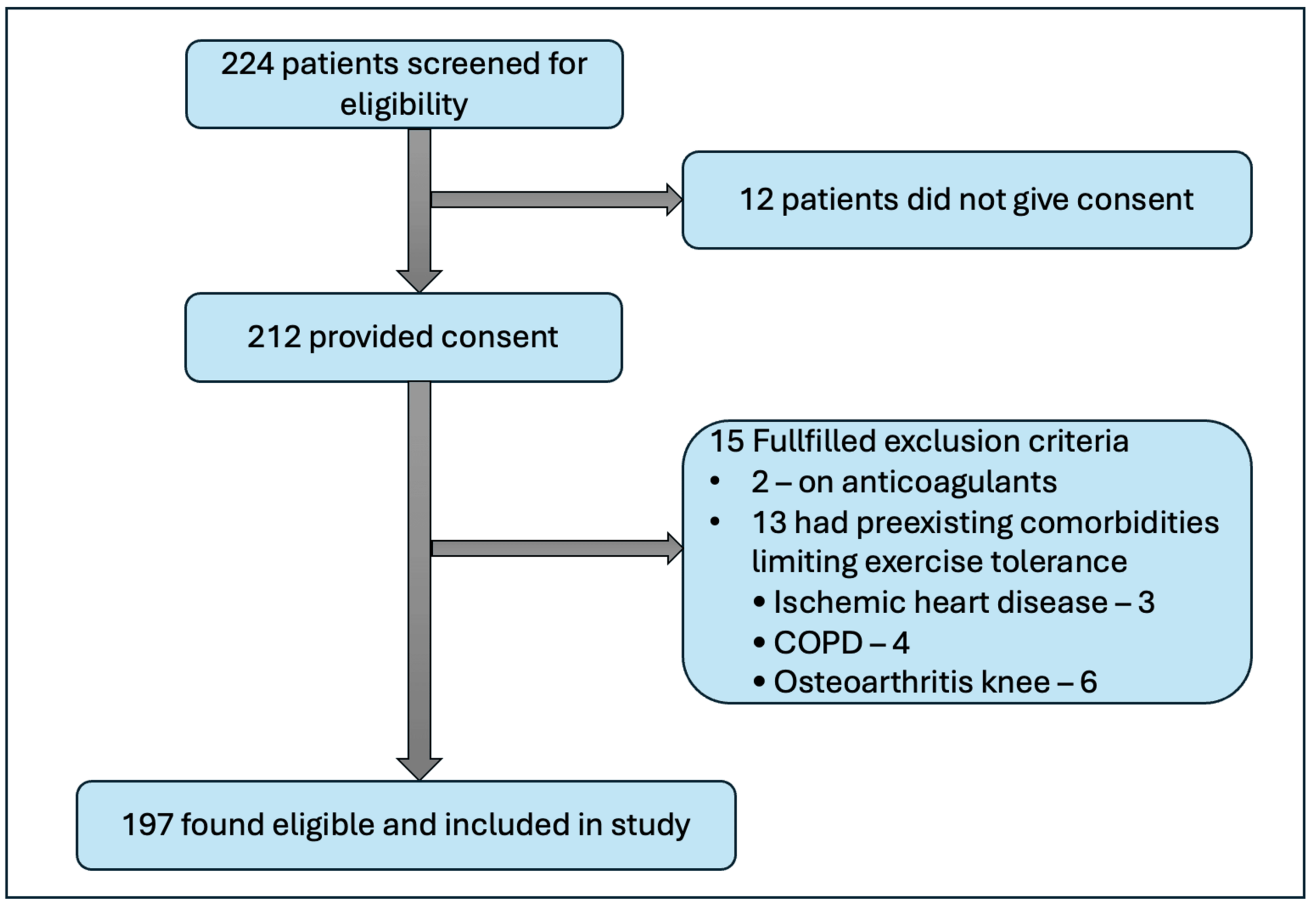
CONSORT diagram illustrating patient screening for eligibility. COPD = chronic obstructive pulmonary disease

A total of 224 consecutive patients coming to the post-COVID-19 clinic were screened for eligibility. In total, 197 were found eligible and included in the study (Figure 1).

Continuous variables were assessed for normality using the Shapiro-Wilk test. The mean age of the study population was 37.3 ± 11.97 years, with 110 females accounting for 55.84% of the study population. The predominant symptom was decreased effort tolerance/easy fatiguability in 151 (76.65%) patients, followed by myalgia/body pain in 72 (36.55%), breathlessness in 67 (34.01%), and cough in 48 (24.37%) (Table 1). More than half of the patients (110, 55.84%) failed the 6MWT. Additionally, 75 (38.07%) patients reported severe symptoms, defined as those interfering with routine daily activities.

**Table 1:** Demographic parameters, clinical features, and the six-minute walk test. SD = standard deviation

Parameter	Total (n = 197)
Age, mean ± SD	37.3 ± 11.97
Gender, n (male/female)	87/110
Symptoms, n (as a proportion of the total population)	
Cough	48 (24.37%)
Decreased effort tolerance/Easy fatiguability	151 (76.65%)
Myalgia/Body pain	72 (36.55%)
Breathlessness	67 (34.01%
Failed six-minute walk test	110 (55.84%)
Severe symptoms	75 (38.07%)

Among baseline laboratory investigations (Table 2), elevated D-dimer was the most frequent abnormality, found in 50 (25.38%) patients, followed by raised fibrinogen in 43 (21.83%) and CRP in 34 (17.26%). Prolonged aPTT and PT/INR were noted in 23 (11.68%) and 21 (10.66%) patients, respectively, while thrombocytopenia was noted in 10 (5.08%) patients.

**Table 2:** Baseline laboratory parameters. CRP = C-reactive protein; PT = prothrombin time; INR = international normalized ratio; aPTT = activated partial thromboplastin time

Parameter	Total (n = 197)
Elevated CRP (>12 mg/mL), n (proportion)	34 (17.26%)
Elevated PT/INR (INR > 1.5), n (proportion)	21 (10.66%)
Elevated aPTT (>35 seconds), n (proportion)	23 (11.68%)
Elevated D-dimer (>500 ng/mL), n (proportion)	50 (25.38%)
Elevated fibrinogen (>400 mg/dL), n (proportion)	43 (21.83%)
Decreased platelet count (<150,000 per mL), n (proportion)	10 (5.08%)

When patients were stratified by symptom severity (Table 3), significantly higher levels of D-dimer and fibrinogen were observed among those with severe symptoms compared with those without (D-dimer: 1,010 ± 428 vs. 247.13 ± 299.11 ng/mL, p = 0.001; fibrinogen: 605 ± 155 vs. 258.94 ± 80.61 mg/dL, p = 0.001). No statistically significant differences were noted for platelet count (p = 0.133), PT (p = 0.061), or aPTT (p = 0.69).

**Table 3:** Association of symptom severity (non-severe vs. severe) with laboratory investigations. SD = standard deviation; PT = prothrombin time; INR = international normalized ratio; aPTT = activated partial thromboplastin time

Parameter	Non-severe symptoms (n = 122)	Severe symptoms (n = 75)	t statistic	P-value
Platelet count (lakhs/mL), mean ± SD	2.79 ± 0.69	2.62 ± 0.83	1.51	0.133
PT (seconds), mean ± SD	13.39 ± 2.62	14.34 ± 4.28	1.89	0.061
INR, mean ± SD	1.21 ± 2.78	1.04 ± 0.31	0.49	0.626
APTT (seconds), mean ± SD	28.98 ± 5.17	27.52 ± 5.17	1.82	0.069
D-dimer (ng/mL), mean ± SD	247.13 ± 299.11	1010 ± 428	5.54	0.001
Fibrinogen (mg/dL), mean ± SD	258.94 ± 80.61	605 ± 155	8.53	0.001

An analysis of biomarker abnormalities and symptom severity (Table 4) demonstrated a strong association between elevated D-dimer and fibrinogen levels and the presence of severe symptoms. Patients with raised D-dimer had a threefold higher risk of severe symptoms (95% CI = 2.18-4.13, p = 0.001), while those with elevated fibrinogen had a 2.34-fold higher risk (95% CI = 1.8-3.05, p = 0.001).

**Table 4:** Association between biomarker abnormalities and severe symptoms. RR = risk ratio; CI = confidence interval; χ² = chi-square

Biomarker	Severe (abnormal)	Severe (normal)	Non-severe (abnormal)	Non-severe (normal)	RR (severe/non severe) abnormal	95% CI	χ²	P-value
D-dimer	34	30	16	117	3	2.18–4.13	23.1	0.001
Fibrinogen	29	35	14	119	2.34	1.8–3.05	60	0.001

At the three-month follow-up, data were available for 190 patients. Improvement was observed in symptom severity, with 37 (19.5%) patients reporting severe symptoms, compared to 75 patients initially. Similarly, the proportion of patients who failed the 6MWT decreased to 49 (25.7%).

A subset of 63 patients with abnormal baseline laboratory parameters underwent repeat investigations after three months to assess trends (Table 5). Persistent abnormalities were most frequently observed in D-dimer (32 (50.79%)) and fibrinogen (30 (47.62%)), while elevations in CRP (4 (6.35%)), aPTT (4 (6.35%)), and PT/INR (2 (3.17%)) were less common. Only 1 (1.59%) patient had persistent thrombocytopenia.

**Table 5:** Investigations after three months. CRP = C-reactive protein; PT = prothrombin time; INR = international normalized ratio; aPTT = activated partial thromboplastin time

Parameter	Total (n = 63)
Elevated CRP (>12 mg/mL), n (proportion)	4 (6.35%)
Elevated PT/INR (INR > 1.5), n (proportion)	2 (3.17%)
Elevated aPTT (>35 seconds), n (proportion)	4 (6.35%)
Elevated D-dimer (>500 ng/mL), n (proportion)	32 (50.79%)
Elevated fibrinogen (>400 mg/dL), n (proportion)	30 (47.62%)
Decreased platelet count (<150,000 per mL), n (proportion)	1 (1.59%)

## Discussion

The present study evaluated coagulation abnormalities and their persistence in patients presenting with long COVID symptoms. The main findings were (i) the most frequent long COVID symptoms were easy fatigability, followed by myalgia, dyspnea, and persistent cough; (ii) elevated D-dimer and fibrinogen levels were observed in 25.38% and 21.83% of patients, respectively, and were associated with severity of symptoms; (iii) at the three-month follow-up, the elevation of D-dimer and fibrinogen persisted in 50.79% and 47.62% of patients, respectively; and (iv) 55.8% of patients failed the 6MWT.

These findings reaffirm that while much is known about the prothrombotic state during acute COVID-19, the persistence of hemostatic abnormalities long after clinical recovery remains underrecognized. The hemostatic system, being dynamic and continuously renewed through hepatic and bone marrow synthesis, typically normalizes following resolution of acute inflammation. Therefore, ongoing dysregulation may suggest either unresolved inflammation or persistent endothelial activation driving a sustained procoagulant milieu [6].

Our findings align with those of Townsend et al., who reported persistent D-dimer elevation in approximately 25% of patients recovering from COVID-19 [7]. Similarly, Kalaivani et al. observed elevated D-dimer in 42% of patients at three months, which decreased to 32% at six months [8]. In our cohort, among those with elevated values initially, D-dimer remained persistently high in 50.79% at three months, suggesting delayed normalization of fibrinolytic activity. Interestingly, Ranucci et al. [6] also reported persistently elevated D-dimer levels among post-COVID-19 individuals; however, they found no significant difference between symptomatic and asymptomatic patients. In contrast, although our cohort consisted exclusively of symptomatic individuals, our findings revealed an association between elevated D-dimer levels and the severity of symptoms.

Both D-dimer and fibrinogen levels were significantly higher in patients with severe symptoms, paralleling trends observed in older patients and those who experienced moderate-to-severe acute disease. The combined elevation of D-dimer and fibrinogen likely reflects a sustained inflammatory and prothrombotic state, as supported by earlier studies demonstrating simultaneous activation of coagulation and incomplete fibrinolysis [9-12]. Interestingly, while CRP normalized in most patients by the three-month mark, D-dimer and fibrinogen levels continued to remain high, underscoring that a hypercoagulable state may persist even after systemic inflammation has subsided.

Several studies have explored this persistent endothelial and coagulation activation. Fan et al [9]. demonstrated significantly elevated D-dimer and Factor VIII levels, with reduced antithrombin activity even 12 months after recovery. Similarly, Gerotziafas et al [10]. and von Meijenfeldt et al. [11]. showed enhanced thrombin generation and impaired fibrinolytic capacity at two to four months post-infection. Pretorius et al [12]. described microclots resistant to fibrinolysis and elevated antiplasmin levels, supporting the hypothesis of ongoing endothelial dysfunction and microthrombus formation. Our findings of persistently high D-dimer and fibrinogen levels in long COVID may corroborate these mechanistic insights.

The paradoxical coexistence of elevated D-dimer and blunted systemic fibrinolysis has been described by Nielsen et al [13]. who proposed that localized pulmonary hyperfibrinolysis, with simultaneous systemic hypofibrinolysis, could explain the persistence of D-dimer despite reduced overall fibrin clearance. This theory could reflect our observation of higher D-dimer levels in patients with persistent dyspnea, suggesting the lungs may be a likely site of ongoing fibrin deposition and degradation.

Functional assessment using the 6MWT further supported this link between coagulation abnormalities and physical limitation. Over half of our patients initially failed the 6MWT, and the majority of these demonstrated elevated D-dimer and fibrinogen levels. At the three-month follow-up, symptom severity and functional status improved markedly, with only 25.7% failing the 6MWT. The observed association between impaired 6MWT performance and elevated coagulation markers may reflect persistent functional limitation in long COVID, although the precise pathophysiological mechanisms underlying this relationship remain uncertain. Similar correlations between prolonged inflammatory and metabolic derangements with musculoskeletal manifestations have been noted, where micronutrient deficiencies were identified as contributors to post-COVID-19 fatigue and myalgia in a comparable cohort [14], highlighting the multifactorial nature of long COVID.

Collectively, these findings suggest that long COVID may represent a chronic, low-grade prothrombotic and endothelial activation syndrome, driven by residual inflammatory mediators, endothelial injury, and possibly immune dysregulation. Primary care physicians are often the first point of contact for individuals experiencing persistent symptoms following COVID-19, placing them in a pivotal position to identify patients at risk of long COVID-related morbidity. Importantly, our findings demonstrate that simple, low-cost tools, such as basic coagulation markers (D-dimer and fibrinogen) and the 6MWT, may serve to stratify symptom severity and functional impairment without the need for advanced monitoring or specialized equipment. The 6MWT is a practical bedside assessment that can be performed in any primary care setting with minimal space and no additional technology, yet it offers valuable insight into cardiopulmonary reserve and exertional intolerance. When used alongside readily available laboratory tests, these tools can enable primary care clinicians to promptly identify patients with possible persistent thrombo-inflammatory activity, prioritize further evaluation, and direct limited healthcare resources toward those who need them most. Integrating such simple screening measures into routine post-COVID-19 follow-up may significantly reduce missed diagnoses, prevent delayed referrals, and, ultimately, decrease long-term morbidity associated with long COVID.

One of the major strengths of this study is its focus on practical, inexpensive, and widely available tools that can be implemented in primary care settings without dependence on advanced diagnostics. This is particularly relevant in low-resource healthcare environments where the majority of long COVID patients initially seek medical attention.

This study has several limitations. Although the sample size was larger than many comparable studies, it remains modest and may limit generalizability. Baseline acute-phase data were unavailable for most patients due to multi-institutional management and lack of unified electronic records. Inflammatory assessment was limited to CRP, and endothelial dysfunction, platelet activation, and cytokine markers such as interleukin-6 were not evaluated, limiting mechanistic interpretation. Symptom severity assessment was based on patient-reported interference with daily activities rather than validated scales such as the modified Medical Research Council dyspnea scale or the Post-COVID Functional Status scale, which may reduce standardization. The observational design and absence of multivariable regression analysis limit causal inference. Repeat laboratory testing was performed only in patients with abnormal baseline parameters; therefore, persistence rates were derived from a pre-selected subgroup, and patients who developed new abnormalities during follow-up may not have been captured. Additionally, anticoagulation therapy in selected patients may have influenced follow-up coagulation parameters. Recruitment from a tertiary post-COVID-19 clinic may have introduced selection bias toward patients with greater symptom burden. Finally, the absence of a control group limits the determination of whether these abnormalities are specific to symptomatic long COVID patients or reflect a broader post-COVID-19 recovery pattern.

## Conclusions

Our study demonstrates that a substantial subset of patients with long COVID exhibit persistent activation of the coagulation cascade, particularly elevated D-dimer and fibrinogen levels, several months after apparent clinical recovery. Simple and inexpensive tools such as D-dimer, fibrinogen, and the 6MWT may provide useful adjunctive information for clinical assessment of long COVID in primary care and resource-limited settings. However, larger multicentric longitudinal studies incorporating inflammatory, endothelial, and imaging-based assessments are required before definitive mechanistic or therapeutic conclusions can be drawn.
